# Translational Research in the Era of Precision Medicine: Where We Are and Where We Will Go

**DOI:** 10.3390/jpm11030216

**Published:** 2021-03-18

**Authors:** Ruggero De Maria Marchiano, Gabriele Di Sante, Geny Piro, Carmine Carbone, Giampaolo Tortora, Luca Boldrini, Antonella Pietragalla, Gennaro Daniele, Maria Tredicine, Alfredo Cesario, Vincenzo Valentini, Daniela Gallo, Gabriele Babini, Marika D’Oria, Giovanni Scambia

**Affiliations:** 1 Department of Translational Medicine and Surgery, Section of General Pathology, Università Cattolica del Sacro Cuore, 00168 Rome, Italy or ruggero.demariamarchiano@policlinicogemelli.it (R.D.M.M.); maria.tredicine@unicatt.it (M.T.); 2 Scientific Direction, Fondazione Policlinico Universitario A. Gemelli IRCCS, 00168 Rome, Italy; alfredo.cesario@policlinicogemelli.it (A.C.); marika.doria@policlinicogemelli.it (M.D.); or giovanni.scambia@unicatt.it (G.S.); 3 Comprehensive Cancer Center—Fondazione Policlinico Universitario A. Gemelli IRCCS, 00168 Rome, Italy; geny.piro@guest.policlinicogemelli.it (G.P.); carmine.carbone@guest.policlinicogemelli.it (C.C.); or giampaolo.tortora@unicatt.it (G.T.); luca.boldrini@policlinicogemelli.it (L.B.); antonella.pietragalla@policlinicogemelli.it (A.P.); gennaro.daniele@policlinicogemelli.it (G.D.); or vincenzo.valentini@policlinicogemelli.it (V.V.); or daniela.gallo@unicatt.it (D.G.); gabriele.babini@policlinicogemelli.it (G.B.); 4 Medical Oncology, Department of Medical and Surgical Sciences, Fondazione Policlinico Universitario A. Gemelli IRCCS, 00168 Rome, Italy; 5 Department of Translational Medicine and Surgery, Section of Oncology, Università Cattolica del Sacro Cuore, 00168 Rome, Italy; 6 Department of Radiology, Radiation Oncology and Hematology, UOC Radioterapia Oncologica, Fondazione Policlinico Universitario A. Gemelli IRCCS, 00168 Rome, Italy; 7 Unità di Medicina Traslazionale per la Salute della Donna e del Bambino, Dipartimento Scienze della Salute della Donna, del Bambino e di Sanità Pubblica, Fondazione Policlinico Universitario A. Gemelli IRCCS, 00168 Rome, Italy; 8 Institute of di Radiology, Università Cattolica Del Sacro Cuore, 00168 Rome, Italy; 9 Dipartimento Universitario Scienze della Vita e Sanità Pubblica, Sezione di Ginecologia ed Ostetricia, Università Cattolica del Sacro Cuore, 00168 Rome, Italy

**Keywords:** omics, personalized medicine, Precision Medicine, high-definition medicine

## Abstract

The advent of Precision Medicine has globally revolutionized the approach of translational research suggesting a patient-centric vision with therapeutic choices driven by the identification of specific predictive biomarkers of response to avoid ineffective therapies and reduce adverse effects. The spread of “multi-omics” analysis and the use of sensors, together with the ability to acquire clinical, behavioral, and environmental information on a large scale, will allow the digitization of the state of health or disease of each person, and the creation of a global health management system capable of generating real-time knowledge and new opportunities for prevention and therapy in the individual person (high-definition medicine). Real world data-based translational applications represent a promising alternative to the traditional evidence-based medicine (EBM) approaches that are based on the use of randomized clinical trials to test the selected hypothesis. Multi-modality data integration is necessary for example in precision oncology where an Avatar interface allows several simulations in order to define the best therapeutic scheme for each cancer patient.

## 1. Introduction

Translational research is a rapidly evolving area of biomedical research that aims to facilitate and speed up the transfer of scientific discoveries into clinical practice. It has emerged as a scientific discipline rather recently, in order to fill the gap between clinical and basic research area. The term “translational research” was first used in the national cancer program of United States in the 1990s and then gradually appeared in academic context and educational programs worldwide. A PubMed bibliographic search, using “translational research” OR “translational medicine” terms in the title/abstract field of manuscripts published up to 2020, resulted in 13,109 records starting from the early 1990s. The number of published scientific papers has constantly climbed each year over the past decades with nearly 85% of articles having been published in the last 10 years.

Barry S. Coller, vice president for Medical Affairs and Professor of the Rockefeller University, defined translational science as “the application of the scientific method to address a health need”. Indeed, although translational research is built on the progress of basic research sharing technologies and skills with it, it is characterized by the primary mission to quickly transform and apply the acquired theoretical knowledge and experimental breakthroughs into new health products and diagnostic/therapeutic tools. Similarly, the reverse flow of information, materials and skills returning to laboratory bench from the clinic is critical for science progression and it should not be overlooked. Indeed, laboratory research is modeled by the continuous comparison with the clinic integrating questions and observations, efficacy data, and molecular mechanisms. On this regard, the Nobel Laureate biologist Sydney Brenner, stressed the importance of failed clinical trials and patients’ unexpected responses as valuable “human experiments” to stimulate new hypotheses that may help refine the route in its next iteration [1].

The advent of Precision Medicine has globally revolutionized the approach of research suggesting a patient-centric vision with therapeutic choices driven by the identification of specific predictive biomarkers of response to avoid ineffective therapies and reduce adverse effects. While conventional medicine is historically designed for the “average patient” with a “one-size-fits-all” approach, the new point of view takes into account individual differences in patients.

The final goal is to obtain the most detailed characterization of each patient identifying genetic and molecular singularities through omics technologies, such as next-generation sequencing platforms, immunohistochemical and flow cytometric analysis, microbiota assessment, proteomics, transcriptomic, and metabolomics.

In addition to the implementation of the most innovative “omics” techniques, the ability to develop predictable, reproducible, and reliable preclinical study models is an essential tool to accelerate the successful incorporation of Precision Medicine into mainstream clinical practice. In the oncology field, for instance, the evolution of research technologies has led to the generation of genetically engineered animal models spontaneously developing tumors, patient-derived xenografts and humanized immune-avatar models in which host immune system is replaced by patient’s cells [2,3,4,5,6]. Thus, precision animal modeling is the link between individualized care in human and advances in animal technologies and genetic manipulation. To fully accomplish their role, precision animal models have to be designed to reflect the variability observed in human cohorts in order to define downstream functional consequences and to discriminate causal from correlative factors at relevant efficiency [7]. These study models give the possibility to carry out multi-level exploration of the effects of genetic variants, environmental exposures, or candidate therapeutic strategies in a way that would be impossible or hard to achieve in human studies.

Finally, the increasing amount of multidimensional data streams coming from omics technologies and digital-sensing devices requires the development of standardized methods of data aggregation and analysis, taking advantage from artificial intelligence with emerging computational techniques, such as machine learning as well as sophisticated cloud computing approaches for data sharing. This review will dissect the different aspects of the present and the future of personalized and translational research, specifically focusing on the rapid evolution of omics approaches and of available technologies, highlighting few initiatives as examples of the ongoing projects, and describing the advantages and the challenges of this new era of Medicine.

## 2. The Evolution of Translational Precision Medicine Research

Although the discipline of Precision Medicine may be considered a relatively young field, the underlying concept is not new and can be found as isolated genial intuitions over the last century. The discovery of blood groups in 1901 by Karl Landsteiner may be accounted as one of the first instances of recognizing differences in patient’s biology and applying a stratification strategy in order to match blood donors with their recipients and improve transfusion safety. However, the predictive ability of science had to wait the development of the surrounding technologic ecosystem to fully show its revolutionary potential.

In the second half of 1950, Friedrich Vogel coined the term “pharmacogenetics” as the study of genetics role in drug response and it has been proposed for the first time that inheritance might explain why many individuals differ in drug efficacy and in adverse reactions susceptibility [8].

A milestone in Precision Medicine evolution has been reached in 1998 with the approval of the first matched drug and diagnostic test for monoclonal antibody trastuzumab in breast-cancer patients overexpressing HER2 protein. Another breakthrough achievement in molecularly-driven therapeutic strategy was the introduction of imatinib for the treatment of chronic myeloid leukemia carrying BCR-ABL1 chromosomal translocation [9].

As the mechanistic knowledge of diseases grew together with technology development, Precision Medicine efforts exponentially increased. The advent of genetic age and the end of Human Genome Project in 2003, involving scientists across six nations to sequence the entire human genome, irreversibly changed healthcare approach.

In 2004, the Food and Drugs Administration (FDA) approved the AmpliChip CYP450 pharmacogenetic test, a microarray that classifies patients according to their cytochrome P450 enzymes to determine drug-metabolizing capacity and select the right patient for the right drug at the right dosage. A few years later, the FDA approved a genetic test for CYP2C9 and VKORC1 to improve the prescription of the anticoagulant warfarin [10].

**Figure 1:**
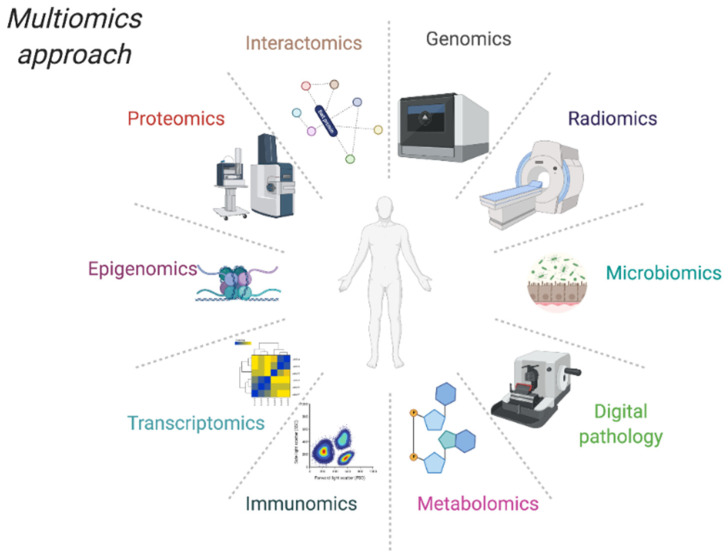
Scheme of “MultiOmics” approach(es). Created with Biorender.com.

In the last years, the increased availability of multigene panel tests, whole genome/exome sequencing, and innovative omic technologies have deeply implemented scientific tools of Precision Medicine (Figure 1). It is now clear that we are at the beginning of an epochal paradigm shift in health care that relies heavily on large-scale collection of biological, radiological, and bioinformatics datasets.

However, to fully apply Precision Medicine vision, a strong institutional support is needed. Many initiatives are underway to create national implementation strategies for Precision Medicine worldwide [11]. For instance, in 2012 started in UK the “100,000 Genomes Project” with the aim to sequence 100,000 genomes of people with cancer or rare diseases and their families and match with National Health Service records and clinical information to uncover new diagnoses and improved treatments for patients. In 2018, Health Secretary Matt Hancock announced that the goal of the project has been achieved. In 2015, the National Institute of Health (NIH) launched a Precision Medicine initiative, named “All of Us Research Program”, to study the genomes and health status of 1 million volunteers with the primary goal of rapidly improving prevention, diagnosis, and treatment of cancer. This is a pioneering participant-centered model aimed to guarantee access to leading edge cancer treatment to all patients. In cancer research field, the era of massive sequencing projects led to unprecedented acceleration toward Precision Medicine. In 2020, the Pan-Cancer Analysis of Whole Genomes (PCAWG) Consortium, an interdisciplinary group of researchers from four continents, presented the most comprehensive and ambitious analysis of cancer genomes so far. This worldwide consortium of scientists carried out integrative analyses of 2658 whole-cancer genomes, matching normal tissues and 1188 transcriptomes across 38 tumor types focusing on cancer drivers [12], non-coding changes [13], mutational signatures [14], structural variants [15], cancer evolution [16], and RNA alterations [17]. Such large-scale initiatives from cooperative groups, pooling together huge numbers of samples and clinical data, is a powerful way to uncover new druggable targets which can be used to tailor therapy to individual patients.

Recently, numerous therapeutic development platforms have been proposed, such as the pan-UK multicenter PRECISION-Panc platform to accelerate the translation of preclinical molecular advances into clinical practice for pancreatic cancer patients finding the right trial for each patient [18,19].

Another ground-breaking initiative comes from the U.S. Pancreatic Cancer Action Network (PanCAN) which is the first pancreatic cancer non-profit organization to develop, sponsor, and lead an adaptive nationwide clinical trial platform, the Precision Promise platform trial (NCT04229004).

## 3. Real-World Data for Translational Research

The rapid technological development that has characterized all the fields of biomedical research in the last years has led to a significant increase of data availability, boosting data dimensionality and inter-actionability.

The validation of new data categories, stemmed out the availability of omics data, opened new frameworks of personalized medicine and translational research.

The number of variables on which the clinical decisional process currently relies in the field of oncology can be considered as a significant example: Abernethy and colleagues have demonstrated that a human is able to simultaneously manage up to five factors in his decision making process (e.g., demographical data like sex or age, signs, and symptoms), while the potential number of decisional variables could rise up several thousand from different knowledge domains (e.g., omics sciences) [20].

This huge amount of data needs to be collected, categorized, and analyzed using appropriate tools and the use of informatics and artificial intelligence has become therefore crucial to support humans in these tasks.

Electronic health record (EHR) archiving systems have rapidly become fundamental tools and it has been demonstrated that healthcare professionals spend two hours of EHR related back office work for each clinical activity hour dedicated to the patient [21].

In addition to the traditional sources of data, there is great interest in data harvested from real life contexts, the so called “real world data” (RWD) that are changing data analysis and interpretation paradigms.

Despite their promising use in research activities, a conclusive definition of RWD is still an object of debate in the scientific community, varying from “data that are not collected in conventional randomized clinical trials”, to “data obtained by any non-interventional methodology that describe what is happening in normal clinical practice” [22].

The European commission has recently released a more comprehensive definition for health RWD, describing them as data collected in medical records, registries, administrative or insurance related databases, or through surveys and mobile applications (accessed on 28 February 2021, https://ec.europa.eu/research/health/pdf/factsheets/real_world_data_factsheet.pdf).

RWD-based translational applications represent a promising alternative to the traditional evidence-based medicine (EBM) approaches that are based on the use of randomized clinical trials to test the selected hypothesis. The RWD approach should not be considered opposite the traditional EBM, but only different from it in terms of collected data quality and dimension, collection methodologies and interpretation.

More specifically, EBM studies have rigid patients’ inclusion and exclusion criteria and aim to answer to a very specific question (e.g., the efficacy of a given treatment on a specific population affected by a single disease). The results of these studies are then summarized in guidelines that support the clinical decision-making process: despite being practical and easy to use tools, these guidelines hardly take into account the different characteristics of the single patients, limiting the impact and the potentialities of a more comprehensive and aware use of all the available data.

The aforementioned characteristics make standard Randomized Control Trial unable to answer the always more complex questions raised by precision and personalized medicine, requiring a paradigm shift in the generation of clinical and translational scientific evidence [23].

Researchers aim therefore to integrate RWD in an innovative conjugation of systems medicine, targeting a more efficacious data governance and enhancing data and knowledge transferability.

However, the comprehensive integration of these data still presents numerous flaws connected to different domains, which are no longer contained in the traditional 4Vs of big data (volume, variety, velocity, and veracity) [24], such as:

- Classification: with ontological inconsistencies at registry, procedural, and research levels.
- Quality: with syntactic (e.g., uterine cancer in a man), semantic (e.g., erroneous meaning assignments), or research (e.g., inconsistent correlations) relevance.
- Privacy and intellectual property.
- Technical: relative to informatics or computational limits.

These limits do not allow to take full advantage of healthcare RWD as a complete research tool, representing a significant obstacle for their introduction in clinical and research practice, either from an authorization, economical or academic perspectives [25,26,27].

The introduction of innovative RWD data management AI-based platforms is therefore strongly needed and will allow a more efficient application of translational-based decisional support systems, personalized approaches and multi-omics predictive models. These tools are able to collect and elaborate previously inconceivable amounts of data, leading clinicians to completely rethink patients’ paths of care, exploring previously unknown correlations among variables relevant to different and apparently not correlated knowledge domains (e.g., patient’s prognosis and the quantitative features of his bioimages) [28].

The informatics architecture of this kind of platform should provide for the continuous interaction of four structural layers, interconnected and interdependent among them [29].

The first layer (computing layer) is represented by hardware and software computational resources.

The following second layer (information layer) is represented by a data catalog and data actionability level, that aims to identify the most appropriate ontological and algorithmic approach, moving from traditional statistics approaches (i.e., regression models), to more advanced machine learning, deep learning, and cognitive analysis applications.

The third layer (user layer) is represented by multidisciplinary working groups in which researchers and clinicians interact with information technologists to run the translational analysis and optimize the applied AI tools [30].

The fourth and last layer (market layer) is oriented towards industrial research partners and stakeholders: synthetic RWD data are exposed for the joint development of models and decisional support systems in protected virtual environments [28,31].

Health data management and interpretation represent for sure one of the most significant and contemporary challenges for all the biomedical sciences and particularly for medicine. New professional figures of clinical data scientists will therefore be needed in the nearest future, open to the introduction and exploration of these innovative research techniques based on the complex AI analysis of translational, clinical, and patient generated RWD.

## 4. Omics Data for Translational Research

Personalized medicine revolutionized disease treatment along with the parallel development of innovative technologies: (i) omics technology for the digitalization of genetic, biological, and morphological characteristics of patient and pathological tissues; (ii) analytic instruments to directly monitor relevant individual or environmental biological and clinical parameters; (iii) technological analysis of big data (e.g., machine learning and artificial intelligence); and (iv) technology of connection and sharing of the data (file systems, Map-Reduce program systems, resilient distributed datasets, etc.).

**Figure 2:**
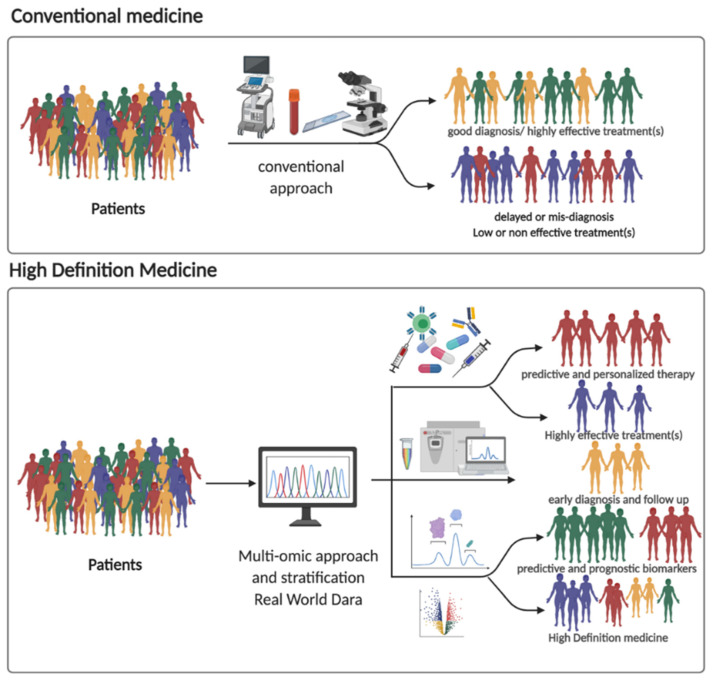
Schematic comparison of high-definition medicine with conventional approaches. Created with Biorender.com.

The widespread use of omics analyses and sensors, together with the ability to acquire huge clinical, environmental, and behavioral information, will lead to the digitalization of the monitoring of people’s health and disease, and to the creation of a global system of real-time management, toward new opportunity for prevention and therapy of the individual person (high-definition medicine, Figure 2).

Further characterization of tissue/systemic dysfunction at a molecular level will enhance our ability to understand, explain, and apply the omics analyses: genomics, epigenomics, transcriptomics, proteomics, interactomics, metabolomics, microbiomics, radiomics, each of these disciplines evaluates different biological and environmental aspects (Figure 1). Moreover, their costs are dramatically decreasing. Although the enormous availability of data, the revolution of the personalized medicine cannot be associated to the Information and Communication Technologies (ICT) instruments or to the ones that have been developed to acquire and analyze data. In fact, personalized medicine is the product of informatics and engineering sciences meeting life sciences. Multi-modality data integration is necessary, for instance, for precision oncology in which an avatar interface is required, meaning that each oncologic patient should have a specific number of simulations to define the best individual therapeutic scheme.

Among all the several existing omics platforms, those for the analysis of nucleic acids are the most developed and have the lowest costs, although they are the most advanced in the validation practices; for this reason, they also are the most applicable in the clinic practice. Sequences of the human genome significantly aided our comprehension of biological processes, even if many of the obtained information still needs to be elucidated and related to the functions of classes of biomolecules, especially proteins. With increasing accessibility to genomic testing and greater understanding of genomic variation on both an individual and worldwide scale, efforts to promote the integration of genomics—and thus the individualization of health care—into health care systems represent a fundamental gain. Biobanks of human germline DNA samples are being used to generate genomic data linked to clinical information from Electronic Health Records (EHRs) in health systems. These biobanks represent a rich resource for the discovery, translationality, and implementation of genomics in medicine. With dense, longitudinal clinical data, her-linked biobanks can boost the study of the natural history of disease, facilitating the implementation of individualized strategies for early detection, prevention, and management of disease. National biobanks are emerging in countries such as the United Kingdom [32], China [33], Japan [34], and others [35].

Structural genomics in the field of cancer basically investigates the three-dimensional structure of all proteins encoded by a genome using computational techniques along with experimental work, resulting in a comparative analysis where different fields of structural biology can be studied simultaneously. Immunomics identifies the interaction of cancer biology with the individual’s immune system [36]. Four main types of tumor-specific antigens are commonly recognized: those encoded by oncogenes, those derived from mutation of any one gene, those differentially expressed only in cancer cells, and those encoded by genes overexpressed in certain types of tumors. Thus, it is possible to determine a genomic profile and to also associate it with the development of a certain humoral immune response [36] or cellular immune response [37,38] in order to obtain an immunomic molecular fingerprint of cancer [39]. Currently in the postgenomic era, the interaction between different omics data (transcriptomics, proteomics, interactomics and metabolomics) introduced a new concept of disease identification and potential integration of omics in the perspective of personalized medicine, the operomic profile.

Precision Medicine, as the ultimate goal of personalized medicine, is a team effort in which different fields of human biology combine to generate a complete picture that can help to dissect the complexity of diseases. Genomics gives important information about the genetic assessment of a human being, but nothing relevant about gene expression (transcriptome) and whether they are functional (proteomics). Proteins are the functional molecules of cells/tissues that control the complex biological pathways (interactome) necessary for health, and whose dysregulation often leads to disease. Furthermore, human diseases produce measurable changes in the human proteome, and most drug targets are proteins [40].

Cancer has paved the way for the introduction of Precision Medicine, and several publications on this topic have demonstrated the potential of proteomics, combined with other omics platforms, such as the 2014 Pioneer 100 Persons Wellness Project [41]. Improved validation methodology will lead to a dramatic increase in the number of approved assays entering the clinic. In this context, interactomics will continue to play an important role, especially in understanding cancer biology and to identify new biomarkers and drug targets [42].

Undoubtedly, a significant hurdle will be the management of big data, deriving from the enormous amount of oncoproteogenomic data that will be generated, and from large heterogeneous datasets of other resources such EHR or data obtained from smartphone apps and personal monitoring devices, the so-called “Avatar of health” [43]. Specific new methods to optimize data collection, storage, cleaning, processing, and interpretation have been and will be developed [44].

The emerging field of digital pathology allowed pathologists to actively contribute to a better understanding of cancer pathogenesis through histo-genomics, the interface between morphology and genomics [45]. Histo-radiomics, the interface between radiology and histology, is another emerging field that integrates radiological imaging with digital pathology images, genomics, and clinical data, providing a more holistic approach to understanding and treating cancer [46].

Similar to the association studies in other fields, the epigenomic wide association study (EWAS) detects epigenetic marks associated with a certain phenotype and, to correct the confounding factors in the data, technical and biological covariates are added to the linear regression models used. Epigenetic profiles can be viewed on appropriate web tools, such as UCSC Genome Browser [47], EpiGenome Browser [48], or coMET [49]. The Cancer Genome Atlas (TCGA) project has produced DNA methylation data for over 10,000 cancer samples [50]. In addition to validating functional roles in cancer etiopathogenesis, epigenetics has also provided useful diagnostic biomarkers and drug targets, specifically among the most promising classes of cancer biomarkers due to their stability, potential reversibility, and ease of access. Some have been approved in non-invasive cancer diagnosis, such as Cologuard, the first test for colorectal cancer (CRC), or more recently the Epi procolon, both assessing DNA methylation [51].

Dynamic profiling of intracellular pathways is a fundamental help in understanding molecular processes related to oncopathogenic processes. As example, Oncobox and other similar approaches were effective in finding numerous biomarkers of biological processes applying the study of interactomics to various aspects of oncology [52].

Metabolomics, still under development in the field of molecular diagnostics, has been particularly used in the study of cancer, achieving promising results, with integration to other platforms [53].

Knowledge about the tumor microbiome has raised many expectations as a helpful potential tool to improve the lives of cancer patients and their response to specific types of cancer drugs [54]. In this context, personalized medicine, targeting the microbiota with different strategies (including nutrition, antibiotic selection, probiotic administration, or fecal microbiota transplantation) will become one of the next frontiers for patients, offering new opportunities with therapies tailored to individual patients [55,56,57].

The personalized medicine revolution comes from the integrative convergence of important developments in systems biology, the “Internet of Things,” and artificial intelligence that will allow us to enter the so-called 6-P medicine era (Predictive, Preventive, Personalized, Participatory, Psychosocial, and Public). It will impact the health status of society by enabling democratized access to comprehensive and personalized health care, healthy lifestyle, through integrative technological and digital (ICT) approaches, combined with ethics and behavioral sciences, and based on Human Avatar (HA), accurate human models, developed and implemented using omics sciences, big data, and advanced imaging.

This is the vision that inspires the Health EU program (under Horizon H2020-FETFLAG-2018-2020) in its vision to provide a Human Avatar (HA) system, composed of two highly interactive components, on the one hand the Digital Human Avatar (DHA, digital models/representations of organs and physiological functions with their underlying molecular network) and on the other hand the Physical Human Avatar (PHA, a component of the HA that combines experimental data from multi-omics, sensory and imaging sources that can characterize multiple human conditions). The accuracy and predictive ability of a DHA and related models are highly dependent on the quality and standards of the datasets and the technological advances that support the PHA. The two vehicles are highly interactive and together form a unique Human Avatar technology that can be individually customized. While most of the digital computing for Human Avatars will be efficiently distributed among, e.g., fog, and cloud computing, this technology will be accessible and usable by all categories of end users through disruptive Avatar-based human-computer interfaces. New generations of Human Avatar User Interfaces (HAUIs) will be developed, with varying levels of system complexity, interaction, configurability, and advanced visualization capabilities, addressing both the professional needs of healthcare professionals and the demands of citizens, including Healthcare Personal Assistant Device (HPAD) feedback loops and advanced Quantified Self (QS) prevention capabilities and services. In addition, in the future, Human Avatars will become ideal user interfaces for mobile healthcare applications and biobehavioral feedback for healthy living (Figure 2).

## 5. GerSom and GENERAtOR Projects: Italian Initiatives

Recently, two wide Italian projects have been proposed to draw new models of translational therapeutic development.

The Fondazione Policlinico Universitario “A. Gemelli” IRCCS coordinates a project aimed at the validation of a gene panel (GerSom) of Alleanza Contro il Cancro (ACC) within a network of laboratories of scientific institutes for research, Hospitalization and Healthcare (IRCCS) afferent to ACC in patients with breast, ovarian and colon cancer (ACC-GerSom project) [58].

This research program aims to study the feasibility of a combined diagnostic process including gene expression quantification and the comprehensive identification of driver and actionable somatic gene alterations in the tumor (for prognostic purposes and definition of the response to therapy), together with the germ line analysis of 172 genes whose pathogenic variants predispose to cancer (CPGs). A further genotyping analysis of ~1,000,000 Single Nucleotide Polymorphisms (SNPs) allows for increased prediction the prediction potential of the genetic cancer predisposition. For each patient carrying a genetic predisposition, the analysis is extended to his/her first-degree relatives in order to organize specific prevention plans for those sharing the cancer predisposition pattern.

The possible benefits for the health care system are the promotion of a national database for the interpretation of the clinical significance of mutations in cancer, the implementation of Clinical Trials for the treatment of patients with specific mutations and the sharing of national guidelines for the management of people with such hereditary cancer predisposition (Precision Prevention).

Increasingly, patients are empowered with a greater awareness of the implications of having a specific mutation. Based on the GerSom project is grafted another collaborative project of the Fondazione Policlinico Universitario A. Gemelli with three other research institutes and an advocacy group of germline mutation carriers, aiming to create knowledge and awareness of the prevention and surveillance processes that hereditary predisposition to cancer involves and to facilitate enrollment in a dedicated clinical trial, to significantly improve social awareness of genetic risk management (project Mutagens).

Another ambitious Italian project is the GEmelli NEtwoRk for Analysis and Tests in Oncology and medical Research (GENERAtOR) research program of the Fondazione Policlinico Universitario “A. Gemelli” IRCCS, (Accessed on 28 February 2021 at https://gemelligenerator.it), which is aimed to offer innovative AI solutions for translational research using the enormous legacy institutional data lake, which is composed by nearly 700 million granular data.

The GENERATOR data analysis multidisciplinary team has developed different AI tools, end user proposals:

A. Mini-bots: software realized for task automation and standardization, such as data recognition and collection, process selection and projection, preliminary data analysis, validation and reporting, or rapid learning solutions, in which the AI tool automatically learns and optimizes its performances during its own activity. These mini-bots are characterized by explainable AI applications, in which explicit algorithms process data whose integrity is guaranteed from the semantic and ontological point of view by the attending researcher. Being explicit algorithms, the human intervention is always possible, and the given output is directly comprehensible for the average scientist-user, granting process transparency, repeatability, and traceability in every phase of the translational analysis.
  Different mini-bots can be realized: one of the most popular examples are: the guardian bot, thought to automatically warn the researchers in case specific events occur (e.g., collection of out of range values); process bot, that identifies deviations from selected guidelines or from the expected behavior of a specific phenomenon; advanced data manager bot that collect and make actionable data of different sources and type (e.g., elastic search and text mining tools that integrate into e-platform lab reports, clinical charts and records, surgical reports, or visits).
B. Avatar: these tools are represented by advanced algorithms, specifically trained to create decisional support systems able to predict clinical outcomes, such as prognosis, treatment related toxicities or complications, therapy results, or diagnostic performances of a specific approach. These Avatars may represent a digital twin of the single patient.
  Avatars may successfully be used in the setup of virtual trials that will for sure boost the potentialities of these approaches.
C. Synthetic data packages: these totally anonymized, General Data Protection Regulation (GDPR) compliant by design, data packages could be used to generate and develop translational and clinical studies in certified and protected virtual environments in which innovative data analysis techniques, coming from knowledge domains other than the traditional biomedical ones, can be successfully applied in the framework of the most fruitful open innovation paradigms.
D. Advanced radiomics and quantitative bio-imaging analysis tools. These image analysis platforms will enrich the value of standard clinical imaging with new decisional variables and translation meaning, thanks to the extraction of certified radiomics features. In this way also the institutional imaging data-lake can be successfully made actionable, flanking the image scientist in both his clinical and research activities [59,60].
E. Informatics solutions aiming to integrate data extracted from portable devices (i.e., fitness bracelets and other types of wearables) in the innovative framework of patient generated RWD, e-health 2.0 clinical trials.

The goal of this project was to enhance treatment personalization, efficiently overlooking the articulated domains of translational research and creating previously unknown synergies among the different data sources, integrating them in the research rationale finding and clinical decision making. The previously described projects are in line with the current research trend for personalized medicine in Europe, where similar ongoing and future initiatives have multiplied (Table 1).

## 6. The CERVGEN Project: A Next Step towards Precision Medicine in Cervical Cancer

Moreover, in the wake of the initiatives described so far, the Fondazione Policlinico Universitario A. Gemelli IRCCS has also recently coordinated a project aimed at incorporating biological information into clinical practice in cervical cancer management. The project has involved an interdisciplinary consortium of health professionals with diverse backgrounds, working in different organizations including Hospital (Fondazione Policlinico Universitario A. Gemelli IRCCS), University (Università Cattolica del Sacro Cuore), and different National Research Centers (ENEA and CNR). Research results have been patented and the scientific paper [61] recognized with the award “ICPerMed-Best Practice in Personalized Medicine-Recognition 2020”.

Using a proteomic approach, integrated with gene expression profiling, the research team has discovered a panel of three protein-coding genes able to predict neoadjuvant chemoradiotherapy treatment outcome, in patients with locally advanced cervical cancer. Importantly, the dataset collected through qPCR analysis of the three genes has been used as a training dataset to implement and optimize a Random Forest algorithm to classify two groups of patients according to their response to therapy. The approach proposed might be easily exploited in the clinical setting to predict the response of new patients, given the qPCR values of gene expression, as obtained from the pretreatment biopsy analysis. As a future perspective, an inexpensive and easy-to-use RNA-based array will be developed allowing patient allocation to personalized treatment procedures, with possibly higher successful rate and significant benefits to both patients and healthcare system.

## 7. Data Privacy/Security

Security standards for omics data in electronic health records (EHRs) have not yet sorted out. So far, data generated by omic tests are collected and protected the same as any other laboratory test results. Although it is reasonable in terms of privacy/security, it could poorly feasible due to the fact that often omic results are too large and too sensitive to store within EHRs; for example, a whole genome sequencing contains about 3 billion base pairs and requires up to 150 gigabytes.

Moreover, the biggest challenge to data privacy in the era of personalized medicine is the fact that there are no absolutes; in fact, the perception of privacy is individual and could change depending on the circumstances; within this context clinical and technical practices, technologies and laws should be very sensitive to multiomic data that are not inherently private just because they disclose genetic or other type of personal information. In other words, the legislation of each country should balance between the individual denied consent to record predictive indicators on a health-alert wristband and the usefulness of these information in the management of patient [62].

An important aspect of the security/privacy issue is also how protected information should be incorporated into HER, solving not only the difficulties of storage as previously explained, but above all addressing the challenge of sensitivity; for example, a whole genome should be strongly protected separating phenotypic information from individual’s demographic data. In this context, the protection processes concern different levels that could be summarize in three phases: (i) the “possession”, that means holding a copy of the data; (ii) the “access”, that regards the permission of consulting data; (iii) the “use”, that implies to formulate or see results derived from the data. Ideally the “data holders” should be minimized, while researchers and providers should have limited access to data, preferably encrypting and anonymizing non-essential details for them. In these regards, federated query across multiple data storage could provide specific responses without having direct access to the data themselves. Some examples are summarized in Table 2.

Therefore, the unmet need of each country/government, in the era of personalized medicine, is the establishment of policies to protect the health data of individuals, in terms of confidentiality, privacy, and security, ensuring at the meantime that the community can take advantage from the scientific development deriving from the open use of data [63].

## 8. Discussion

This vision of a paradigm shift in healthcare is only possible through engineering advances in sensing, computing, communication, and low energy cloud/fog technologies, along with new modeling and computational approaches to leverage big data, such as artificial intelligence and neuromorphic systems, and such as the design and development of components of a specific data infrastructure and subclass of the Internet of Things called the Internet of Healthcare (IoH). The IoH will have integrated rules for security, privacy, and ethics, and will serve as a reference for future e-Health.

Human Avatars are a practical solution that aims to improve people’s health and disease burden and that can reduce the inefficiency of health care systems due to (a) fragmentation of care, (b) adoption of therapeutic strategies and medications which disregard individual genetic determinants resulting in poor cost effectiveness, and (c) lack of active participation in disease prevention and management and poor patient compliance. The basic idea is to facilitate the collaborative work of doctors by providing them with individualized and holistic data and to empower and actively involve each individual in managing their own health risks. Both these measures should promote wellness and reduce inequalities and costs in health care systems.

Although there has long been a need and recognition that translational effectiveness from research to care requires the systematic access and integration of research and health care at a large scale and possibly across institutions and countries, identifying reliable tools to integrate datasets remains one of the most daunting challenges faced by the field. Combining omics data into a single model is also fraught with controversy, and to date, one of the unmet needs is the identification of a consensual and robust methodology [64].

In more general terms, one of the main obstacles to data integration is data comparability and consistency. Biomedical data are often heterogeneous, incomplete, and inaccurate by nature. Even the task of obtaining and integrating electronic health records (EHRs) across hospitals, within a country, has proven to be much more complex than expected, even in the most advanced health systems [65]. Initiatives are underway in Europe to establish robust platforms for collecting and sharing standardized data, such as DIFUTURE in Germany [66] (10.3414/ME17-02-0022) and other similar initiatives in individual EU states, such as Alleanza contro il Cancro in Italy [67]. Compared to the United States, one Europe advantage seems to be the ability to generate networks such as Data Integration Centers that could collect and process data at national and supranational levels.

The introduction of machine learning within artificial intelligence (AI) approaches seems particularly well suited to address these challenges, although even within this field the amount of original data and its proper standardization remain of paramount importance [68,69]. Moreover, on several levels beyond the obvious privacy concerns, artificial intelligence poses serious concerns, including adversarial attacks [70], for which appropriate ethical boundaries would need to be implemented [71].

Thus, the new era of big data in medicine offers several new challenges, as well as great opportunities, to improve the health of humankind, not only for rich nations, but also for developing countries. Patients, doctors, clinical lab technicians, and researchers would need to gain new knowledge, and most importantly interact and acquire new mind-sets and perspectives, leading to a completely overhauled healthcare ecosystem [72]. Clinicians would need to engage in more pervasive interaction with clinical laboratory technicians and researchers to have a more effective interaction. In addition, patients would be required to become more disease aware, with the ultimate goal of removing barriers that still prevent the delivery of the best treatments to patients, leading to a form of “participatory” medicine between patients, clinicians, and their community [73]. Along these lines, the entire matrix of data, information, knowledge, and wisdom (DIKW) has been proposed for personalized medicine, in which “smart, empowered patients” can take a primary and leading role in their own health, taking greater responsibility for their own health and well-being [74].

## 9. Conclusions

To realize these exciting prospects, it is critical to address the challenges that underlie safe and effective technological innovation in this area by developing consensus standards through the identification and discussion of priority short- and long-term challenges. Changes in cultural and educational paradigms are needed at various levels, including the shift to data sharing. Only if the research community is conceptually ready to share and integrate data across the globe, will the AI tools be able to meet high expectations and contribute positively to the advancement of biomedical research.

## Figures and Tables

**Table 1:** EU supported initiatives concerning activities on personalized medicine, in alphabetical order. Source: CORDIS, https://cordis.europa.eu/en (accessed on 5 March 2021). Query: content type = ‘personalized medicine’ AND ‘initiatives’ AND ‘ongoing’.

EU-Code	Acronym	Title	Start Date	End Date
951724	B1MG	Beyond 1M Genomes	1 June 2020	31 May 2023
715772	BabyVir	The role of the virome in shaping the gut ecosystem during the first year of life	1 April 2017	30 September 2022
777090	Back-UP	Personalized Prognostic Models to Improve Well-being and Return to Work After Neck and Low Back Pain	1 January 2018	30 April 2021
115974	BEAt-DKD	Biomarker Enterprise to Attack DKD	1 September 2016	31 August 2021
821511	BIOMAP	Biomarkers in Atopic Dermatitis and Psoriasis	1 April 2019	31 March 2024
679586	BUMP BETTER	Understanding the metaphysics of pregnancy	1 April 2016	31 March 2021
876362	CHARM	Challenging environments tolerant Smart systems for IoT and AI	1 June 2020	31 May 2023
825775	CINECA	Common Infrastructure for National Cohorts in Europe, Canada, and Africa	1 January 2019	31 December 2022
821520	ConcePTION	Building an ecosystem for better monitoring and communicating of medication safety in pregnancy and breastfeeding: validated and regulatory endorsed workflows for fast, optimized evidence generation	1 April 2019	31 March 2024
765158	COsMIC	COmbatting disorders of adaptive immunity with Systems MedICine	1 January 2018	31 December 2021
949850	DCUBATION	Redefining the term ‘Incubation period’ using large-scale digital data	1 November 2020	31 October 2025
806968	EHDEN	European Health Data and Evidence Network	1 November 2018	30 April 2024
724115	ENABLE	European Academy for Biomedical Science	1 July 2016	30 June 2021
824160	EnTimement	ENtrainment and synchronization at multiple TIME scales in the MENTal foundations of expressive gesture	1 January 2019	31 December 2022
779282	ERA PerMed	ERA-Net Cofund in Personalized Medicine	1 December 2017	30 November 2022
806948	ESCulab	European Screening Centre; Unique Library for Attractive Biology	1 December 2018	30 November 2023
964333	EU-Africa PerMed	Building links between Europe and Africa in personalized medicine	1 February 2021	31 January 2025
825843	EU-STANDS4PM	A European standardization framework for data integration and data-driven in silico models for personalized medicine	1 January 2019	31 December 2021
952103	EuCanImage	A European Cancer Image Platform Linked to Biological and Health Data for Next-Generation Artificial Intelligence and Precision Medicine in Oncology	1 October 2020	30 September 2024
825903	euCanSHare	An EU-Canada joint infrastructure for next-generation multi-Study Heart research	1 December 2018	30 November 2022
824753	FETFX	Stimulating effects of Future and Emerging Technologies through communication and outreach	1 January 2019	30 June 2021
101017549	GenoMed4ALL	Genomics and Personalized Medicine for all though Artificial Intelligence in Hematological Diseases	1 January 2021	31 December 2024
945334	Gravitate-Health	Empowering and Equipping Europeans with health information for Active Personal Health Management and Adherence to Treatment	1 November 2020	31 October 2025
823939	GreenX4Drug	Green Enantioselective Halogenation for Drug Discovery and Manufacture	1 April 2019	31 March 2023
116026	HARMONY	Healthcare Alliance for Resourceful Medicines Offensive against Neoplasms in HematologY	1 January 2017	31 December 2021
957532	HEART.FM	Maximizing the Therapeutic Potential of Music through Tailored Therapy with Physiological Feedback in Cardiovascular Disease	1 November 2020	30 April 2022
874694	IC2PerMed	Integrating China in the International Consortium for Personalized Medicine	1 January 2020	31 December 2023
731366	ICPerMed	Secretariat for the International Consortium for Personalized Medicine (IC PerMed)	1 November 2016	30 April 2021
964197	ICPerMed	Secretariat for the International Consortium for Personalized Medicine (ICPerMed)	1 March 2021	29 February 2024
853981	IDEA-FAST	Identifying Digital Endpoints to Assess FAtigue, Sleep and acTivities in daily living in Neurodegenerative disorders and Immune-mediated inflammatory diseases	1 November 2019	30 April 2025
831514	Immune-Image	Specific Imaging of Immune Cell Dynamics Using Novel Tracer Strategies	1 October 2019	30 September 2024
101016775	INTERVENE	International consortium for integrative genomics prediction	1 January 2021	31 December 2025
826121	iPC	Individualized Pediatric Cure: Cloud-based virtual-patient models for precision pediatric oncology	1 January 2019	31 May 2023
825821	iReceptor Plus	Architecture and tools for the query of antibody and t-cell receptor sequencing data repositories for enabling improved personalized medicine and immunotherapy	1 January 2019	31 December 2022
681043	JPsustaiND	Coordination Action in support of the sustainability and globalization of the Joint Programming Initiative on Neurodegenerative Diseases	1 November 2015	31 October 2021
101017453	KATY	Knowledge At the Tip of Your fingers: Clinical Knowledge for Humanity	1 January 2021	31 December 2024
678304	LEASP	Learning spatiotemporal patterns in longitudinal image data sets of the aging brain	1 September 2016	31 August 2021
732592	Lifebrain	Healthy minds from 0-100 years. Optimizing the use of European brain imaging cohorts	1 January 2017	30 June 2022
777377	LITMUS	Liver Investigation: Testing Marker Utility in Steatohepatitis	1 November 2017	31 October 2022
965286	MAESTRIA	Machine Learning Artificial Intelligence Early Detection Stroke Atrial Fibrillation	1 March 2021	28 February 2026
873262	MAGELIA	A disruptive Magnetically Enhanced Library preparation platform for Next Generation Sequencing	1 August 2019	31 July 2021
820820	MOBILISE-D	Connecting digital mobility assessment to clinical outcomes for regulatory and clinical endorsement	1 April 2019	31 March 2024
893699	MODIRen	Integrative metabolomics and genomics analysis for the development of markers of inherited kidney diseases: a personalized medicine approach	4 January 2019	3 January 2023
806975	NECESsITY	NEw Clinical Endpoints in primary Sjögren’s Syndrome: an Interventional Trial based on stratifYing patients	1 January 2019	31 December 2024
724334	NOSUDEP	A Wearable Electronics Approach To Reduce Mortality in Epilepsy	1 September 2017	28 February 2023
825410	ONCOBIOME	Gut OncoMicrobiome Signatures (GOMS) associated with cancer incidence, prognosis and prediction of treatment response.	1 January 2019	30 June 2024
693124	ONOFF	Perception of voices that do not exist: Tracking the temporal signatures of auditory hallucinations	1 September 2016	31 December 2021
946050	PACE	Platform for Rapid Development of Personalized Nanomedicine Drug Delivery Systems	1 September 2020	28 February 2022
101016851	PANCAIM	Pancreatic cancer AI for genomics and personalized Medicine	1 January 2021	31 December 2024
951773	PerMedCoE	HPC/Exascale Centre of Excellence in Personalized Medicine	1 October 2020	30 September 2023
115976	PHAGO	Inflammation and AD: modulating microglia function focusing on TREM2 and CD33	1 November 2016	31 October 2021
716079	PREDICT	PREcision medicine Drug combination testing In neuroblastoma organoids to guide Clinical Trials	1 March 2017	28 February 2022
754425	PROMINENT	Personalized Medicine in Diabetic Chronic Disease Management	1 September 2017	31 August 2022
754907	R-LiNK	Optimizing response to Li treatment through personalized evaluation of individuals with bipolar I disorder: the R-LiNK initiative	1 January 2018	31 December 2022
115902	RADAR-CNS	Remote Assessment of Disease and Relapse in Central Nervous System Disorders	1 April 2016	31 March 2022
825746	RECODID	Integrated human data repositories for infectious disease-related international cohorts to foster personalized medicine approaches to infectious disease research	1 January 2019	31 December 2022
825812	REGIONS4PERMED	interregional coordination for a fast and deep uptake of personalized health	1 November 2018	30 April 2023
857491	REMODEL	Research models in infection, cancer and regeneration: replacement and translation	1 November 2019	31 October 2022
801540	RESCUE	Local Training Network on REgenerative medicine and Stem Cell technology in UtrEcht	1 June 2018	31 May 2023
847912	RESCUER	RESistance under Combinatorial treatment in ER+ and ER- breast cancer.	1 January 2020	31 December 2024
825046	SAPHIRE	Securing Adoption of Personalized Health in REgions	1 December 2018	31 May 2022
874556	SINO-EU-PerMed	Widening Sino-EU policy and research cooperation in Personalized Medicine	1 January 2020	December 2023
826117	Smart4Health	Citizen-centered EU-EHR exchange for personalized health	1 January 2019	28 February 2023
875534	SOPHIA	Stratification of Obese Phenotypes to Optimize Future Obesity Therapy	1 June 2020	31 May 2025
733112	SPIDIA4P SPIDIA	Standardization of generic Pre-analytical procedures for In-vitro DIAgnostics for Personalized Medicine	1 January 2017	30 June 2021
825884	SYNCHROS	SYNergies for Cohorts in Health: integrating the ROle of all Stakeholders	1 January 2019	31 December 2021
733100	SYSCID	A Systems medicine approach to chronic inflammatory disease	1 January 2017	31 March 2022
730994	TERRINet	The European Robotics Research Infrastructure Network	1 December 2017	30 November 2021
821283	TransBioLine	Translational Safety Biomarker Pipeline: Enabling development and implementation of novel safety biomarkers in clinical trials and diagnosis of disease	1 February 2019	31 January 2024
831458	Trials@Home	Center of Excellence—Remote Decentralized Clinical Trials	1 September 2019	31 August 2024
668353	U-PGx	Ubiquitous Pharmacogenomics (U-PGx): Making actionable pharmacogenomic data and effective treatment optimization accessible to every European citizen	1 January 2016	30 June 2021
820755	VALUE-Dx	The value of diagnostics to combat antimicrobial resistance by optimizing antibiotic use	1 April 2019	31 March 2023
826421	VirtualBrainCloud	Personalized Recommendations for Neurodegenerative Disease	1 December 2018	30 November 2022
824128	VIRTUALTIMES	Exploring and Modifying the Sense of Time in Virtual Environments	1 January 2019	31 December 2022

**Table 2:** Partial examples of data sources and website addresses subdivided for countries. Source: CORDIS, https://cordis.europa.eu/en, accessed on 5 March 2021. Query: content type = ‘personalized medicine’ AND ‘initiatives’ and ‘ongoing’. All URLs in the Table have been accessed on 5 March 2021.

Data Sources	
Country	Website Addresses
Worldwide	https://www.scilifelab.se/news/33-milion-to-large-scale-genomic-research/ https://www.genomeweb.com/informatics/million-european-genomes-alliance-proposed-response-obamas-precision-medicine-initiative#.YD9gyZNKg0o https://www.icpermed.eu/app/login http://www.fudan-pgx.org/premedkb/index.html#/home https://www.cancerimagingarchive.net/
Europe	https://project-iasis.eu/ https://upgx.eu/https://precise4q.eu/
UK	https://www.gov.uk/government/news/dna-mapping-to-better-understand-cancer-rare-diseases-and-infectious-diseases https://www.cancerresearchuk.org/about-us/cancer-news/news-report/2018-02-21-100000-genomes-project-hits-halfway-milestone https://www.genomicsengland.co.uk/
Norway	https://www.nordforsk.org/search/precision%20medicine https://nos-m.org/news/nordic-common-strengths-and-future-potential-in-the-field-of-personalised-medicine https://www.heartbioportal.com/ https://neic.no/tryggve/ https://www.forskningsradet.no/om-forskningsradet/portefoljer/ https://www.ntnu.no/biobanknorge https://helse-midt.no/vart-oppdrag/prosjekter/ehelse/helseplattformen http://www.genomics.no/ https://www.uib.no/praksisnett
Denmark	https://www.dtu.dk/english/news/2017/07/danish-reference-genome-now-mapped https://www.healthcaredenmark.dk/news/new-danish-genome-centre-for-research-on-personalized-medicine.aspx https://lundbeckfonden.com/soeg?s=personalized+medicine
Finland	https://www.riigiteataja.ee/en/eli/531102013003/consolide https://julkaisut.valtioneuvosto.fi/handle/10024/74459 https://stm.fi/julkaisu?pubid=URN:ISBN:978-952-00-3575-4 https://www.sitra.fi/en/topics/well-being-data/ https://www.finngen.fi/en/about https://www.eurekalert.org/pub_releases/2017-12/uoh-fag121917.php https://www.fimm.fi/en/research/grand-challenge-programmes/finnish-genome-sequencing-preventive-health-care/sisu-project https://www.businessfinland.fi/en/for-finnish-customers/services/programs/personalized-health-finland
US	http://www.personalizedmedicinecoalition.org/ https://pm.jh.edu/
Canada	https://www.genomecanada.ca/
Asia	https://genomeasia100k.org/

## Data Availability

No new data were created or analyzed in this study. Data sharing is not applicable to this article.
